# Managing prostate cancer after proctocolectomy and ileal pouch-anal anastomosis: feasibility and outcomes of single-port transvesical robot-assisted radical prostatectomy

**DOI:** 10.1007/s00345-024-05051-9

**Published:** 2024-06-04

**Authors:** Adriana M. Pedraza, Ethan L. Ferguson, Roxana Ramos-Carpinteyro, Carter Mikesell, Jaya S. Chavali, Nicolas Soputro, Nima Almassi, Christopher Weight, Emre Gorgun, Jihad Kaouk

**Affiliations:** 1 Glickman Urological and Kidney Institute, Cleveland Clinic, 9500 Euclid Ave, Q10, Cleveland, OH 44195 USA; 2 Colorectal Surgery, DDSI, Cleveland Clinic, Cleveland, OH USA

**Keywords:** Prostate cancer, Proctocolectomy and ileal pouch-anal anastomosis, Single-port transvesical robot-assisted radical prostatectomy, Robot-assisted radical prostatectomy

## Abstract

**Introduction:**

Patients with proctocolectomy and ileal pouch-anal anastomosis (PC-IPAA) face unique challenges in managing prostate cancer due to their hostile abdomens and heightened small bowel mucosa radiosensitivity. In such cases, external beam radiation therapy (EBRT) is contraindicated, and while brachytherapy provides a safer option, its oncologic effectiveness is limited. The Single-Port Transvesical Robot-Assisted Radical Prostatectomy (SP TV-RARP) offers promise by avoiding the peritoneal cavity. Our study aims to evaluate its feasibility and outcomes in patients with PC-IPAA.

**Methods:**

A retrospective evaluation was done on patients with PC-IPAA who had undergone SP TV-RARP from June 2020 to June 2023 at a high-volume center. Outcomes and clinicopathologic variables were analyzed.

**Results:**

Eighteen patients underwent SP TV-RARP without experiencing any complications. The median hospital stay was 5.7 h, with 89% of cases discharged without opioids. Foley catheters were removed in an average of 5.5 days. Immediate urinary continence was seen in 39% of the patients, rising to 76 and 86% at 6- and 12-month follow-ups. Half of the cohort had non-organ confined disease on final pathology. Two patients with ISUP GG3 and GG4 exhibited detectable PSA post-surgery and required systemic therapy; both had SVI, multifocal ECE, and large cribriform pattern. Positive surgical margins were found in 44% of cases, mostly Gleason pattern 3, unifocal, and limited. After 11.1 months of follow-up, no pouch failure or additional BCR cases were found.

**Conclusion:**

Patients with PC-IPAA often exhibit aggressive prostate cancer features and may derive the greatest benefit from surgical interventions, particularly given that radiation therapy is contraindicated. SP TV-RARP is a safe option for this group, reducing the risk of bowel complications and promoting faster recovery.

**Supplementary Information:**

The online version contains supplementary material available at 10.1007/s00345-024-05051-9.

## Introduction

Proctocolectomy and ileal pouch-anal anastomosis (PC-IPAA) is a successful strategy for reducing the risk of malignancy in patients with ulcerative colitis and familial adenomatous polyposis. It is the preferred procedure when medical treatments have proven ineffective for ulcerative colitis, and selected cases of Crohn’s colitis, offering an improved quality of life by preserving the anal sphincter mechanism and the natural passage of stool over a permanent end ileostomy [1]. Managing prostate cancer in the context of a previous PC-IPAA presents significant difficulties. Firstly, these patients have hostile abdomens due to the multi-stage nature of the PC-IPAA procedure, which involves total colectomy, proctectomy, IPAA with loop ileostomy, and ileostomy reversal [2]. Secondly, the ileal mucosa of the pouch exhibits increased radiosensitivity compared to the rectum, making external beam radiotherapy (EBRT) a contraindicated treatment option [1]. Lastly, recent evidence has revealed a strong association between pelvic inflammation and adverse pathologic features in prostate cancer, [3] adding an extra layer of complexity to the management. As a result, while brachytherapy monotherapy has been proposed as a safer alternative in these cases, considering its effectiveness is restricted to a specific spectrum of the disease, surgery may still offer the best chance for a cure in this complex patient group.

Single-port transvesical robot-assisted radical prostatectomy (SP TV-RARP) has emerged as a promising option for patients with hostile abdomens. This technique proposes a regionalized approach, ensuring the peritoneal cavity remains untouched. In the United States, robotic surgeries have become the leading choice for managing prostate cancer, surpassing open or laparoscopic approaches. However, the existing literature on RARP in patients with prior PC-IPAA is limited, with only a few reported cases, all utilizing multiport and transperitoneal approaches [4, 5]. In this context, our study is the first to integrate both the SP system and the TV approach. By exploring this novel technique, we aim to broaden the available options for patients with PC-IPAA history and pave the way for potentially improved outcomes in their prostate cancer management.

## Methods

### Study population

Between June 2020 and June 2023, a cohort of 18 consecutive patients with prostate cancer and a history of PC-IPAA underwent SP TV-RARP. These surgeries were performed by an experienced surgeon at a tertiary care center. The data were collected prospectively within an IRB-approved database, and a retrospective analysis was performed.

### Variables and definitions

Extracapsular extension (ECE) was defined as the presence of a tumor expanding beyond the borders of the gland, and it was categorized as either focal or nonfocal, in accordance with the Epstein method [6]. Cribriform architecture was defined as a contiguous sheet of malignant epithelial cells with multiple visible glandular lumina, clearly noticeable at low magnification [7]. Margins were classified as positive when cancerous tissue was in direct contact with the inked surface of the prostatectomy specimen.

Patient follow-up assessments were conducted at 6 weeks, 3 months, 6 months, and 12 months post-surgery.

Erectile function was marked by a SHIM score of ≥ 17, and continence by the use of zero or one safety pad. Perioperative complications were noted using the Clavien-Dindo classification, [8] and readmission was defined as any condition needing admittance within the initial 30 days after the surgery. Biochemical recurrence (BCR) was identified when the PSA level reached ≥ 0.2 ng/mL, measured within the 6–13-week timeframe post-surgery, and validated by a second PSA level exceeding 0.2 ng/mL [9].

### Surgical technique

**Fig. 1:**
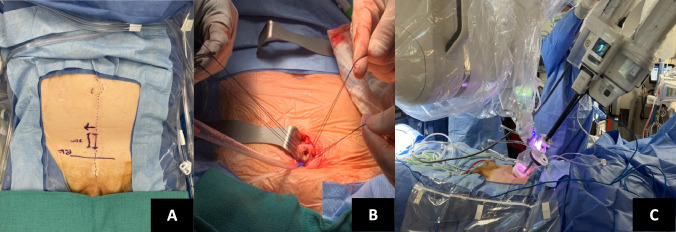
**A** Incision closer to the pubic symphysis and approximately 1 cm away from the previous scar. **B** Cystostomy after stay suture placement. **C** Single-port access and docking

Our SP TV-RARP technique has been described previously [10]. In brief, the patient is positioned supine with minimal Trendelenburg. A midline suprapubic incision of 3 to 4 cm is made for access. Once the anterior rectus fascia is opened, the rectus abdominis muscles are separated, the bladder is identified, and four stay sutures are placed. A 2 cm vertical cystotomy is performed, and the da Vinci SP access port (Intuitive Surgical Inc., Sunnyvale, CA, USA) is introduced (Fig. 1). After locating the ureteral orifices, a semicircular incision is made at the posterior bladder neck between the 5 and 7 o’clock positions. The dissection continues through the retrotrigonal layer, identifying the vas deferens and seminal vesicles, and then proceeds posteriorly between the pouch and the prostate, reaching the apex. Subsequently, an incision is made at the bladder neck anteriorly. The endopelvic fascia is opened, puboprostatic ligaments are transected, and the dorsal venous complex is controlled. The urethra is identified and transected. Pedicle control is achieved using bipolar energy, and the nerve-sparing is performed in a retrograde manner. If PLND is needed, traction is applied to the bladder neck in a lateral direction, exposing the pelvic sidewall to perform a limited dissection. Upon completing the posterior reconstruction, the urethrovesical anastomosis (UVA) is carried out continuously, progressing bilaterally from the 6 o’clock to the 12 o’clock position [11].

### Surgical considerations in patients with PC-IPAA

**Fig. 2:**
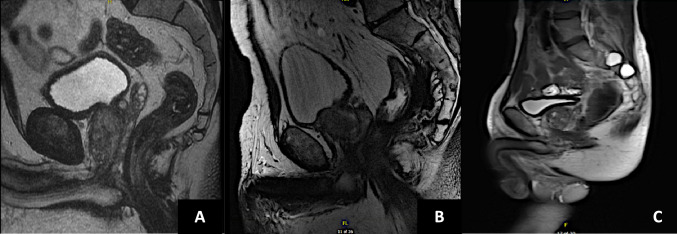
Examples of anatomical variations in patients with prior PC-IPAA. The sagittal view of the mpMRI is used to plan the access, taking into account the relationship of the bladder with the pubic symphysis and the bowel (**A**–**C**). In these scenarios, the incision for patient **C** would be closer to the symphysis compared to patients (**A** and **B**), with a more pronounced trendelenburg position and bladder distension necessary before cystostomy

Effective preoperative planning is essential to determine the optimal access during surgery. In these cases, the suprapubic incision is strategically placed closer to the pubic symphysis, deviating from the conventional two fingerbreadths above, as in regular patients. Additionally, in instances of prior laparotomy, the incision is meticulously positioned approximately 1 cm away from the existing scar, as shown in Fig. 2. Employing some Trendelenburg positioning is recommended to facilitate bowel displacement.

In these cases, a significant challenge arises from the presence of fibrosis and scar tissue between the prostate/SVs and the ileal pouch, complicating the posterior dissection—especially when there is concurrent seminal vesicle involvement by the tumor. To mitigate the risk of complications, we minimize the use of monopolar energy during this step and opt for cold scissors and bipolar energy for dissection. Additionally, we utilize the Remotely Operated Suction Irrigation System (ROSI) for retraction, suction, and occasionally blunt dissection. Lastly, during UVA, we decrease the pressure of the pneumovesicum (< 8 mm Hg) to alleviate tension while performing the anastomosis (Video).

### Statistical analysis

The descriptive characteristics of the patients were presented using means and standard deviations (SD), along with medians and interquartile ranges (IQR) for continuous variables. Categorical variables were presented in terms of frequencies and percentages. All statistical analyses were conducted with STATA^®^ 18 (StataCorp, College Station, Texas).

## Results

### Patients’ characteristics

Table 1 displays the baseline characteristics of the patients. The average age was 65.9 years, and the majority (83%) were diagnosed with intermediate-risk prostate cancer.

**Table 1:** Baseline patients’ characteristics and perioperative Outcomes

Baseline characteristics	SP transvesical (*N* = 18)
Age, years, mean, range	65.9 (55–75)
Race, N (%)	
Caucasian	17 (94.4)
African American	1 (5.6)
BMI, kg/m^2^, median (Q1, Q3)	28 (24–30)
ASA, median (Q1, Q3)	3 (3–3)
Charlson comorbidity index, median (Q1, Q3)	4 (4–6)
Time since IPAA surgery, years, median (Q1, Q3)	23 (10.9–31.9)
Prostate size, grams, median (Q1, Q3)	49.3 (42–62)
Preoperative PSA, ng/ml, median (Q1, Q3)	6.6 (5.2–10.5)
ISUP GG, N (%)	
1	1 (5.6)
2	9 (50)
3	6 (33.2)
4	1 (5.6)
5	1 (5.6)
NCCN risk category, N (%)	
Low-very low	1 (5.6)
Intermediate—favorable	9 (50)
Intermediate—unfavorable	6 (33.3)
High risk/very high risk	2 (11.1)
Preoperative SHIM, mean (range)	18 (5–23)
Preoperative IPSS, mean (range)	6 (2–13)
Time from biopsy to surgery, months, median (Q1, Q3)	2.9 (1.6– 6.6)
Biopsy approach	
Transperineal	8 (44.4)
Transrectal	9 (50)
Transgluteal	1 (5.6)
Follow-up, months, median (Q1, Q3)	11.1 (9.9–15.8)
Perioperative outcomes	
Console time, minutes, mean, range	130 (105–154)
Estimated blood loss, cc, median (Q1, Q3)	94 (50–100)
Lymphadenectomy, N (%)	2 (11)
Briganti nomogram 2019, median (IQR)	3 (1–7)
Nerve sparing, N (%)	
Bilateral	10 (54)
Unilateral	4 (22)
None	4 (22)
Intraoperative complications, N (%)	0
Length of hospitalization, hours—median (Q1, Q3)	5.7 (4.6–21.2)
Patient encounter type, N (%)	
Inpatient	2 (11)
Outpatient	16 (89)
Pain scale at discharge—median (Q1, Q3)	3 (2–5)
Opioid prescription at discharge, N (%)	2(11)
Postoperative complications, N (%)	2 (11)
Foley catheter duration, days—mean, range	5.5 (3–9)
Pathology ISUP GG, N (%)	
2	14 (77.8)
3	3 (16.7)
5	1 (5.5)
Margins, N, (%)	
Negative	10 (56)
Unifocal, limited, and Gleason pattern 3	4 (22)
Unifocal, non-limited, and Gleason pattern 3	2 (11)
Multifocal, limited, and Gleason pattern 3	1 (5.5)
Multifocal, non-limited, and Gleason pattern 4	1 (5.5)
Lymph node yield—median (Q1, Q3)	4 (4–4)
Pathologic staging, N (%)	
pT2	9 (50)
pT3a	6 (33.3)
pT3b	3 (16.7)
Continence, N (%)	
Immediate	7/18 (39)
6-week	8/18 (44)
3-month	10/17 (59)
6-month	13/17 (76)
12-month	12/14 (86)
Postop PSA, N (%)	
BCR	2 (11)
Undetectable	16 (89)

*BMI* Body mass index, *ASA* American society of anesthesiology, *IPAA* ileal pouch anal anastomosis, *PSA* prostate specific antigen, *ISUP GG* International society of urological pathology grade group, *NCCN* national comprehensive cancer network risk, *SHIM* sexual health inventory for men, *IPSS* international prostate symptom score

### Perioperative and functional outcomes

The median time from PC-IPAA to SP TV-RARP was 23 years. Despite significant adhesions between the IPAA and the prostate and seminal vesicles, the SP TV-RARP was successfully performed in all patients without any pouch violation, open conversion, additional ports, or bowel complications. After a median hospital stay of 5.7 h, 89% of patients were discharged without opioids, and Foley catheters were removed in an average of 5.5 (range 3–9) days. Our protocol for the trial of void does not include a cystogram. It involves instilling roughly 300 cc of saline into the bladder, followed by evaluation of micturition upon catheter removal. Notably, 39% of cases achieved immediate urinary continence, with 76 and 86% being fully continent at 6 and 12 months after surgery, respectively (Table 1). Regarding erectile function, among the cases with a baseline SHIM score ≥ 17 and excluding those who required postoperative ADT (5/18), we found that 40% of the patients who completed the 12-month follow-up remained potent after the intervention (2/5).

There were no major postoperative complications in our series. Minor complications were reported in 2 patients with Clavien-Dindo I and II events, respectively.

### Oncologic outcomes

The oncologic characterization of this population holds significant importance. Half of the cohort had non-organ confined disease, with 27.8% (5/18) being upstaged from stages IIB and IIC to stage IIIB. Moreover, 3 out of 18 patients (16.6%) were upgraded in the final pathology, transitioning from ISUP Grade Group (GG) 1 to GG2, GG2 to GG3 with tertiary Gleason 5, and GG2 to GG5, respectively. Intraductal carcinoma (IDC) was detected in 22.2% (4/18) and cribriform architecture in 66.6% (12/18) of the cases. Among patients with pT3 disease, 4 out of 9 showed focal ECE, while the remaining 5 out of 9 had nonfocal ECE (Table 1).

Two patients had detectable PSA post-surgery. The first, initially diagnosed with GG 2 prostate cancer, a PSA of 13 ng/ml, and a PIRADS 4 lesion on mpMRI, showed pT3b, GG 2 with cribriform features and non-focal ECE on final pathology. Despite a negative postoperative prostate-specific membrane antigen positron emission tomography (PSMA PET), the PSA level remained elevated at 2.04 ng/ml. The second patient, with a history of a failed prostate ablation, was diagnosed with ISUP GG3. The mpMRI identified a left anterior apical peripheral zone PI-RADS 5 lesion, and the F-18 Fluciclovine PET-CT confirmed no systemic involvement. The final pathology revealed a pT3b disease, non-focal ECE, IDC, and a cribriform architecture. The postoperative PSA remained detectable at 0.49 ng/ml, and a PSMA PET revealed suspicious lymphadenopathy. After consultation with the institutional tumor board, he underwent ePLND, confirming involvement in 4 of the 15 lymph nodes. Both patients are currently receiving systemic treatment.

Even though the percentage of positive surgical margins (PSMs) was elevated (8/18, 44%), these were mostly Gleason pattern 3, unifocal, and limited (< 3 mm). It is noteworthy that over 60% of patients exhibiting PSMs had high-risk features in the final pathology. With a median follow-up of 11.1 (9.9–15.8) months, no pouch failure or additional cases of BCR have been identified.

## Discussion

Managing prostate cancer in patients with a history of PC-IPAA presents substantial challenges. These prior complex abdominal surgeries in patients undergoing RARP increase the risk of bowel perforation, obstruction, and conversion to open surgery [12, 13]. Moreover, the augmented radiosensitivity of the small bowel makes EBRT unsuitable for management. While brachytherapy alone is not associated with long-lasting radiation-induced pouch toxicity, [14] only patients with low risk and favorable intermediate-risk prostate cancer may have an oncologic benefit from this approach. Furthermore, its application is restricted by factors such as prostate size, bladder outlet obstruction, or prior transurethral resection of the prostate. As a consequence, brachytherapy may not constitute the optimal treatment choice in this scenario.

An additional consideration lies in understanding the potential drivers for the development of aggressive prostate cancer in cases with PC-IPAA. Recently, Chakravarty et al. found higher rates of adverse pathology and BCR in patients treated with RARP in whom pelvic inflammation was encountered during surgery. These patients had increased expression of interleukin-6 (IL-6) and STAT3 within the tissue as well as elevated systemic levels of pro-inflammatory cytokines [3]. Importantly, the IL‑6/STAT3 signaling pathway has been recognized for its involvement in prostate cancer progression [15, 16]. This phenomenon suggests a more aggressive phenotype stemming from alterations induced in the tumor microenvironment as a consequence of inflammation. In accordance with the aforementioned, we observed a high prevalence of adverse features within our cohort. The rate of upstaging/upgrading stood at 33%, similar to contemporary series (ranging from 24.8% to 45%) [17, 18]. Furthermore, most ECE cases presented a non-focal extension. Although the final pathology of 77.8% of patients displayed ISUP GG 2, cribriform architecture and IDC were identified in 66% and 22% of the specimens, respectively. Overall, these observations correlate with the risk of disease progression. For instance, multiple studies have established that the extent of ECE independently influences both BCR-free survival and progression-free survival [19–22]. Likewise, as highlighted by Ma et al. unfavorable histopathological features, specifically IDC and cribriform architecture, further increase the risk of failure [23]. The authors demonstrated that those patterns were present in 84.4% of ISUP GG 2 prostate cancer patients with metastatic disease, in contrast to 38% among control cases without metastasis. Another factor to consider is the potential for delayed diagnosis and intervention in patients with PC-IPAA. This delay inherently increases the likelihood of disease progression. Therefore, the occurrence of aggressive forms of prostate cancer should not be deemed unexpected in this population.

Radical prostatectomy has demonstrated its feasibility when performed by experienced surgeons with a high caseload. In 2010, Umbreit et al. examined 16 patients with PC-IPAA who underwent open retropubic radical prostatectomy (RP), underscoring the successful completion of all cases, positive functional outcomes, and the absence of long-term postoperative pouch-related complications. The study revealed durable oncologic benefits, with three cases of BCR, two local recurrences, and no systemic progression or deaths attributed to prostate cancer at a mean follow-up of 5.7 years (0.3 to 14.3 years) [24]. Subsequently, in 2014 and 2020, Leapman et al. [4] and Chen et al. [5] independently proved the feasibility of multiport transperitoneal RARP in a total of three cases, each performed without any reported intraoperative or immediate postoperative complication. Meanwhile, Uchino et al. provided insights into the long-term outcomes of 30 patients who underwent RP using either open or robotic approaches.Their findings unveiled a 10-year pouch survival rate of 85.7%, along with favorable oncologic outcomes—overall survival and disease-free survival of 88.5 and 89.7%, respectively [25]. Based on this data while acknowledging the demanding nature of the procedure, RP appears to offer the most promising potential for managing the disease in this group of patients.

The introduction of the SP surgical system has driven a transition towards outpatient surgeries, a reduction in opioid use, and expedited catheter removal after RP [26–28]. Recently, Kaouk et al. developed the SP TV-RARP, further refining the regionalization of the procedure [10]. In this study, we have demonstrated the versatility of the technique. By avoiding peritoneal cavity violation, SP TV-RARP reduces the potential for intestinal injury and promotes faster recovery of bowel function. Remarkably, every case within our cohort was successfully conducted without pouch violation, conversion to open surgery, use of additional ports, or intraoperative complications. Moreover, the benefit of SP TV-RARP extends to the immediate urinary continence rate, reported to be as high as 75% within 48 h of catheter removal [10]. It’s important to consider that patients with PC-IPAA may potentially experience underlying pelvic floor muscle dysfunction due to neural injury at the time of proctocolectomy [29, 30]. This can lead to delayed functional recovery, a factor that should also be taken into account when deciding upon the surgical technique to be offered.

We acknowledge the limitations of our study, which is retrospective and involves a small sample size. Due to the recent introduction of this technique, the follow-up period for our patients was relatively short. Additionally, all cases of SP TV-RARP were performed by an experienced robotic surgeon, potentially impacting the generalizability of the outcomes, especially in these complex cases. Lastly, our technique itself has certain constraints in terms of the extent of PLND feasible through a TV approach, which is typically limited to the obturator fossa. Therefore, an extraperitoneal approach might be preferred for cases requiring ePLND.

## Conclusion

SP TV-RARP is a safe and well-suited option for patients with prior PC-IPAA. Its primary advantage lies in establishing a safer route to access the prostate, effectively reducing the risk of bowel injury and minimizing the extent of lysis of adhesions. Furthermore, SP TV-RARP promotes ambulatory surgeries, reduces the need for opioids, shortens the time to catheter removal, and contributes to an earlier restoration of urinary continence. These perioperative gains will have to be gauged against long-term cancer outcomes with additional follow-up. To the best of our knowledge, our study represents the largest cohort of RARP in patients with PC-IPAA. It stands as the first to propose both the single-port system and the transvesical approach in this context, providing a compelling alternative for managing patients with hostile abdomens and potentially aggressive prostate cancer.

## Supplementary Information

Below is the link to the electronic supplementary material.

Supplementary file1 (MOV 418966 KB)

## Data Availability

We hereby confirm that our data is available for review upon request to the principal investigator as per the required statement for journal publication.
